# The household economic burden of non-communicable diseases in 18 countries

**DOI:** 10.1136/bmjgh-2019-002040

**Published:** 2020-02-11

**Authors:** Adrianna Murphy, Benjamin Palafox, Marjan Walli-Attaei, Timothy Powell-Jackson, Sumathy Rangarajan, Khalid F Alhabib, Alvaro Jr Avezum, Kevser Burcu Tumerdem Calik, Jephat Chifamba, Tarzia Choudhury, Gilles Dagenais, Antonio L Dans, Rajeev Gupta, Romaina Iqbal, Manmeet Kaur, Roya Kelishadi, Rasha Khatib, Iolanthe Marike Kruger, Vellappillil Raman Kutty, Scott A Lear, Wei Li, Patricio Lopez-Jaramillo, Viswanathan Mohan, Prem K Mony, Andres Orlandini, Annika Rosengren, Ismail Rosnah, Pamela Seron, Koon Teo, Lap Ah Tse, Lungiswa Tsolekile, Yang Wang, Andreas Wielgosz, Ruohua Yan, Karen E Yeates, Khalid Yusoff, Katarzyna Zatonska, Kara Hanson, Salim Yusuf, Martin McKee

**Affiliations:** 1 London School of Hygiene and Tropical Medicine Faculty of Public Health and Policy, London, UK; 2 Population Health Research Institute, Hamilton Health Sciences and McMaster University, Hamilton, Ontario, Canada; 3 Department of Cardiac Sciences, King Fahad Cardiac Center, College of Medicine, King Saud University, Riyadh, Saudi Arabia; 4 Dante Pazzanese Institute of Cardiology, São Paulo, Brazil; 5 Department of Health Management, Faculty of Health Sciences, Marmara University, Istanbul, Turkey; 6 Department of Physiology, University of Zimbabwe, Harare, Zimbabwe; 7 Independent University, Dhaka, Bangladesh; 8 Institut universitaire de cardiologie et de pneumologie de Québec, Quebec City, Ontario, Canada; 9 Department of Medicine, University of the Philippines Manila, Manila, Philippines; 10 Eternal Heart Care Centre and Research Institute, Jaipur, India; 11 Department of Community Health Sciences, Aga Khan University, Karachi, Pakistan; 12 School of Public Health, Post Graduate Institute of Medical Education and Research, Chandigarh, India; 13 Isfahan Cardiovascular Research Center, Cardiovascular Research Institute, Isfahan University of Medical Sciences, Isfahan, the Islamic Republic of Iran; 14 Department of Neurology, Northwestern University Feinberg School of Medicine, Chicago, Illinois, USA; 15 Africa Unit for Transdisciplinary Health Research, North-West University, Potchefstroom, South Africa; 16 Health Action by People, Trivandrum, Kerala, India; 17 Faculty of Health Sciences, Simon Fraser University, Vancouver, British Columbia, Canada; 18 State Key Laboratory of Cardiovascular Disease, Fuwai Hospital, National Center for Cardiovascular Diseases, University Teknologi MARA, Beijing, China; 19 FOSCAL, Medical School, Universidad de Santander (UDES), Bucaramanga, Colombia; 20 Dr. Mohan's Diabetes Specialities Centre & Madras Diabetes Research Foundation, Chennai, India; 21 St John's Medical College and Research Institute, Bangalore, India; 22 ECLA Foundation, Santa Fe, Argentina; 23 Department of Molecular and Clinical Medicine, Sahlgrenska Academy, University of Gothenburg and Sahlgrenska University, Gothenburg, Sweden; 24 Community Health Department, Faculty of Medicine, UKM Medical Centre, Kuala Lumpur, Malaysia; 25 Facultad de Medicina, Universidad de La Frontera, Temucu, Chile; 26 JC School of Public Health and Primary Care, Faculty of Medicine, The Chinese University of Hong Kong, Hong Kong; 27 School of Public Health, University of the Western Cape, Bellville, Western Cape, South Africa; 28 State Key Laboratory of Cardiovascular Disease, Fuwai Hospital, National Center for Cardiovascular Diseases, Chinese Academy of Medical Sciences and Peking Union Medical College, Beijing, China; 29 Department of Medicine, University of Ottawa, Ottawa, Ontario, Canada; 30 Department of Medicine, Queen's University, Kingston, New Hampshire, Canada; 31 UiTM, Selayang, Selangor and UCSI University, Cheras, Kuala Lumpur, Malaysia; 32 Department of Social Medicine, Wroclaw Medical University, Wroclaw, Poland

**Keywords:** health economics, health insurance, cardiovascular disease, health systems, diabetes

## Abstract

**Background:**

Non-communicable diseases (NCDs) are the leading cause of death globally. In 2014, the United Nations committed to reducing premature mortality from NCDs, including by reducing the burden of healthcare costs. Since 2014, the Prospective Urban and Rural Epidemiology (PURE) Study has been collecting health expenditure data from households with NCDs in 18 countries.

**Methods:**

Using data from the PURE Study, we estimated risk of catastrophic health spending and impoverishment among households with at least one person with NCDs (cardiovascular disease, diabetes, kidney disease, cancer and respiratory diseases; n=17 435), with hypertension only (a leading risk factor for NCDs; n=11 831) or with neither (n=22 654) by country income group: high-income countries (Canada and Sweden), upper middle income countries (UMICs: Brazil, Chile, Malaysia, Poland, South Africa and Turkey), lower middle income countries (LMICs: the Philippines, Colombia, India, Iran and the Occupied Palestinian Territory) and low-income countries (LICs: Bangladesh, Pakistan, Zimbabwe and Tanzania) and China.

**Results:**

The prevalence of catastrophic spending and impoverishment is highest among households with NCDs in LMICs and China. After adjusting for covariates that might drive health expenditure, the absolute risk of catastrophic spending is higher in households with NCDs compared with no NCDs in LMICs (risk difference=1.71%; 95% CI 0.75 to 2.67), UMICs (0.82%; 95% CI 0.37 to 1.27) and China (7.52%; 95% CI 5.88 to 9.16). A similar pattern is observed in UMICs and China for impoverishment. A high proportion of those with NCDs in LICs, especially women (38.7% compared with 12.6% in men), reported not taking medication due to costs.

**Conclusions:**

Our findings show that financial protection from healthcare costs for people with NCDs is inadequate, particularly in LMICs and China. While the burden of NCD care may appear greatest in LMICs and China, the burden in LICs may be masked by care foregone due to costs. The high proportion of women reporting foregone care due to cost may in part explain gender inequality in treatment of NCDs.

**Key questions**

**What is already known?**

- Worldwide, there is low use of drugs for secondary prevention of cardiovascular disease and hypertension, and there is a gender and socioeconomic gap in use of treatment.
- Research has suggested that out-of-pocket expenditure for non-communicable disease (NCD) healthcare can impose a significant burden on households but highlighted scarcity of research from sub-Saharan Africa, Latin America and the Middle East.

**What are the new findings?**

- Households with NCDs in lower middle income countries (LMICs) spend more on healthcare and are at greater risk of catastrophic expenditure and impoverishment than households without after adjustment for potential confounders.
- An alarmingly large number of individuals with NCDs report not taking prescribed medicines for NCDs due to cost, particularly among women in low-income countries (LICs).

**What do the new findings imply?**

- Insufficient progress has been made towards international goals, and we are far from achieving financial risk protection for people with NCDs, particularly in LMICs and LICs.
- While the burden of NCD care may appear greatest in LMICs, the burden in LICs may be disguised by foregone care due to costs, with women likely most dramatically affected.

## Background

In September 2018, the United Nations (UN) held the Third High Level Meeting on the Control of Non-Communicable Diseases (NCDs). The purpose of the meeting was to track progress towards four commitments made in the 2014 UN Outcome Document on NCDs.^1^ Among these was a commitment to ‘strengthen and orient health systems to address the prevention and control of NCDs… through people-centred primary health care and universal health coverage (UHC)’, echoing the UN Sustainable Development Goal 3, Target 8, to achieve UHC for all. Progress towards this commitment will be judged by an indicator assessing whether member states are providing ‘drug therapy and counseling for eligible persons at high risk to prevent heart attacks and strokes’.^1^

Evidence collected prior to 2014 highlighted why these commitments were so urgently needed.^2^ This showed low use of drugs for secondary prevention of cardiovascular disease (CVD; the leading cause of morbidity and mortality among NCDs) and hypertension (HTN; the most common risk factor) worldwide, and these drugs were unavailable and unaffordable to many people in lower income countries (LICs).^3–6^ Out-of-pocket (OOP) costs for counselling and drug therapy for CVD and related conditions such as HTN, angina or diabetes imposed a significant economic burden on households, driving some into poverty and, in many cases, were a reason for not seeking or adhering to care.^7–11^ The evidence also suggested a gender gap in secondary treatment of CVD in countries at all levels of development, with women less likely to take proven effective treatment than men.^12^ It highlighted how the poor were least likely to obtain treatment^13^ and the devastating impact on them of OOP costs.^14^ Finally, it revealed the scarcity of research on this topic from some regions in lower middle income countries (LMICs), in particular sub-Saharan Africa, Latin America and the Middle East.^2^

Has the situation improved since the 2014 UN commitments were made? Since 2014, the Prospective Urban and Rural Epidemiology (PURE) study has collected data on OOP expenditure for NCDs in 18 countries at all levels of development, making it the most recent and comprehensive dataset on this topic. We set out to estimate the burden of OOP costs borne by households containing patients with HTN and NCDs, and whether these costs are barriers to accessing healthcare.

## Methods

### The PURE Study

This paper presents secondary analysis of data from the PURE Study. PURE is a large ongoing international cohort study of NCD incidence, mortality and risk factors^15^ in individuals from urban and rural communities in 21 countries, with data needed for this analysis available from 18 countries: Canada, Sweden, Brazil, Chile, Malaysia, Poland, South Africa, Turkey, China, the Philippines, Colombia, Iran, the Occupied Palestinian Territory (OPT), Bangladesh, India, Pakistan, Zimbabwe and Tanzania.

Data collection in PURE has been described in detail elsewhere.^15^ Briefly, in each country, communities were selected to achieve a mix of rural and urban populations while ensuring feasibility of data collection (eg, processing blood samples) and long-term follow-up. Households were selected to be representative of the sociodemographic composition of communities, and the sociodemographic characteristics and death rates of the samples of the first 17 participating countries were similar to national populations.^16^ Further details on the representativeness of the PURE cohort are included as online supplementary appendix 1. Within each selected household, all individuals aged 35–70 years were eligible to participate. Baseline data collection, using a standardised questionnaire including information on sociodemographic characteristics and health status, occurred between 2005 and 2014, depending on when the country joined the PURE Study.^15^ The round of follow-up data collection that included health expenditures began in 2014, and the year in which follow-up data were completed in each country is shown in online supplementary appendix 2. 15 17 18

10.1136/bmjgh-2019-002040.supp1 Supplementary data

### Study design

#### Household-level analysis: catastrophic spending and impoverishment

Our objective was to compare the economic burden of direct OOP health expenditure in households with NCDs, HTN and households with neither.

NCD households were defined as those with at least one member with CVD (defined as self-reported history of stroke, heart attack, angina, other CVD at baseline or follow-up), diabetes (self-report of diagnosis or being prescribed treatment at follow-up), cancer (self-reported history of any cancer at baseline or follow-up), respiratory diseases (self-reported history of asthma or chronic obstructive pulmonary disease at baseline or follow-up) and kidney disease (self-reported history of kidney disease or treatment at follow-up only). Households with HTN only were defined as those with at least one member who self-reported diagnosis or being prescribed treatment at follow-up. Baseline HTN or diabetes was not used to define our sample as there was a small percentage of people in each country (from 0.1% to 4.5%) without NCDs who self-reported HTN or diabetes at baseline but not at follow-up (online supplementary appendix 3). These people were not included in our definition of having an NCD or HTN (but were included in the non-NCD/HTN group if their household had no other NCDs). Overall, loss to follow-up in the PURE Study was 4.4%. The number of households that either did not respond to the expenditure questionnaire, or were lost to follow-up, by country income group, is shown in online supplementary appendix table 4. Of the 70 346 households that completed follow-up, our analytical sample includes only those households (n=51 920) that completed the expenditure questionnaire (73.8%). The number of NCD households in this analysis was n=17 435, of HTN households it was 11 831 and non-NCD/HTN households numbered 22 654.

The PURE follow-up questionnaire included a module on household expenditure based on the WHO World Health Survey instrument.^19^ Expenditures reported for the last month included consultation fees paid to doctors/nurses, or traditional/alternative healers, diagnostic or laboratory tests, medication and ambulance costs. Those reported for the last year included costs associated with overnight stays in a hospital/health facility or a long-term facility (reported for the last year). Expenditures reimbursed by insurance were not included nor were insurance premiums. All reported annual expenditures were converted to monthly amounts and to adjusted to a common base year and currency for which exchange rates were available for all included countries (2011 international dollar).

We compared NCD households and HTN-only households with non-NCD households on the following measures: (1) mean OOP health expenditure as a percentage of effective income; (2) proportion of households experiencing catastrophic healthcare spending; and (3) proportion of households impoverished. We defined these measures using definitions used by the WHO European Region and described in the 2017 WHO/World Bank (WB) UHC Global Monitoring Report^20^ and elsewhere.^21^ Specifically, effective income is defined as per capita total expenditure remaining after deducting an estimated amount for basic needs (estimated as per capita spending on food, housing and utilities of those households in the 25th–35th percentile of total per capita expenditure).^21 22^ Catastrophic healthcare spending was defined as cases where per capita OOP health expenditures were ≥40% of per capita effective income. Impoverished households were defined as those whose total per capita expenditure gross of per capita OOP healthcare expenditure was above the basic needs line, but total per capita expenditure net of OOP per capita healthcare expenditure was below it. See online supplementary appendix 5 for additional technical details on impoverishment. These definitions were selected to ensure maximum comparability of our study findings with other external estimates given that these are the most widely used definitions globally. Prior to calculating these measures, outlier per capita household total and healthcare expenditures were ‘top-coded’ (ie, converted to the value of the 99th percentile of expenditure for that category).^23^

A crude comparison of the economic burden of OOP costs in NCD households, HTN-only households and non-NCD/HTN households would ignore other drivers of healthcare expenditure, such as the proportion of elderly people living in the household. To account for this, we employed multilevel multivariable regression, adjusting for the potential influence of community (as a random effect), country and the following household characteristics (as fixed effects): age composition (proportion >60 years of age, proportion <5 years of age), gender composition, household size, highest education level attained in the household (primary/none/unknown vs secondary vs tertiary), proportion of the household employed, whether any of the NCD or HTN patients were women and the highest degree of physical impairment caused by the NCD (a continuous measure based on the sum of seven binary questions regarding the effect of the disease on the patients’ ability to use their fingers to grasp, walk without a cane/walker, bend down to pick up objects, read or resolve small objects on a plate, see a person from across a room, speak and hear conversations). We compared the risk difference in the percentage of effective income spent on healthcare, the percentage of households experiencing catastrophic spending and the percentage of households experiencing impoverishment, adjusted for the above household characteristics, between NCD and HTN-only households compared with non-NCD/HTN households. We hypothesised that risk increased with condition severity (ie, from non-NCD/non-HTN to HTN-only to NCD) and conducted regression-based tests for linear trend.

#### Individual-level analysis: financing sources and cost as a barrier to treatment

Individuals with NCDs and HTN in PURE were asked about challenges they faced in adhering to treatment due to cost and about sources of financing of health expenditure. We estimated the individual-level self-reported prevalence of not taking prescribed medicines due to cost (‘In the past 12 months, was there a time when you did not take prescribed medicines due to cost?’), self-reported difficulties paying for healthcare (‘In the last 12 months have you experienced difficulty paying for doctor’s fees/medications/diagnostic fees/hospital bills?’) and the use of different sources of financing (eg, using savings, borrowing money and selling assets) to pay for health expenditures (‘In the last 12 months, how did you usually pay for medical/health care costs?’), among individuals with NCDs (n=21 934) and HTN (n=17 512).

All results are presented by country income groupings, according to World Bank classifications^20^: high-income countries (HICs: Canada and Sweden), upper middle income countries (UMICs: Brazil, Chile, Malaysia, Poland, South Africa and Turkey), LMICs (the Philippines, Colombia, India, Iran and the OPT) and low-income countries (LICs: Bangladesh, Pakistan, Zimbabwe and Tanzania). Where means are presented, they are calculated across all individuals within the country income category. China, an UMIC, is presented separately as it has a much larger sample size than any other group of countries. Other country-specific results can be found in online supplementary appendix 6. We also conducted a sensitivity analysis using regional grouping (South Asia: India, Pakistan and Bangladesh; Canada/Sweden; South America: Brazil, Chile and Colombia; China; South East Asia: Malaysia and the Philippines; Middle East: Saudi Arabia, Iran, United Arab Emirates (UAE) and Turkey; and sub-Saharan Africa: South Africa, Tanzania and Zimbabwe) to see if the different grouping makes a difference to the interpretation of results from the country income group analysis. These results can be found in online supplementary appendix 7. All analyses were conducted using Stata V.15.

### Patient and public involvement statement

Patients were not involved in this study.

## Results

Table 1 shows descriptive statistics of households in our sample by country income group. On average, NCD and HTN-only households tended to be older than other households, and in every country income group except for LICs, a higher proportion of the household was employed. NCD households in LMICs, LICs and in China tended to be better educated than the HTN-only and non-NCD/HTN households, consistent with findings from some other NCD and NCD risk factors (eg, physical inactivity) studies in these settings.^24–26^

**Table 1:** Household descriptive statistics, analytical sample of NCD, HTN-only and non-NCD/HTN households: PURE Study

	High income			Upper middle income			Lower middle income			Low income			China		
	NCD	HTN-only	Non-NCD/HTN	NCD	HTN-only	Non-NCD/HTN	NCD	HTN-only	Non-NCD/HTN	NCD	HTN-only	Non-NCD/HTN	NCD	HTN-only	Non-NCD/HTN
	n=2760	n=1507	n=3379	n=5459	n=3513	n=4769	n=3429	n=1948	n=4418	n=515	n=409	n=1231	n=5249	n=4446	n=8794
Mean of mean household age (years)	50.2	49.0	41.1	41.9	40.6	35.9	37.8	37.7	31.6	30.9	32.0	29.9	48.0	45.3	41.2
95% CI	49.3 to 51.1	48.0 to 50.0	40.4 to 41.8)	39.9 to 43.9	38.7 to 42.5	34.5 to 37.3	37.0 to 38.7	36.8 to 38.6	30.8 to 32.5	29.0 to 32.9	30.6 to 33.3	28.4 to 31.4	46.7 to 49.3	44.1 to 46.5	39.9 to 42.6
P value for difference in means*	0.0000			0.0000			0.0000			0.0001			0.0000		
Mean household size	2.2	2.2	2.5	3.7	3.8	3.9	3.8	3.7	4.3	5.7	5.3	5.2	2.9	3.0	2.9
95% CI	2.1 to 2.3	2.1 to 2.3	2.4 to 2.6	3.3 to 4.1	3.3 to 4.2	3.6 to 4.2	3.6 to 4.1	3.5 to 3.9	4.0 to 4.5	4.8 to 6.6	4.5 to 6.0	4.9 to 5.6	2.8 to 3.0	(2.8 to 3.1)	(2.8 to 3.1)
P value for difference in means*	0.0000			0.0069			0.0000			0.1117			0.1064		
Mean proportion of female household members (%)	53.1	52.3	52.4	53.1	53.0	53.1	50.4	51.5	50.2	53.7	54.0	52.4	51.1	50.5	50.7
95% CI	51.7 to 54.5	51.0 to 53.7	51.2 to 53.6	51.5 to 54.7	51.6 to 54.4	51.7 to 54.5	49.1 to 51.6	50.0 to 53.0	49.2 to 51.2	(0.1 to 57.4	51.0 to 56.9	50.3 to 54.6	50.3 to 52.0	(49.4 to 51.6)	(49.7 to 51.7)
P value for difference in means*	0.4624			0.9744			0.0941			0.1415			0.2687		
Mean proportion of employed household members (%)	91.5	92.0	85.3	59.7	60.0	55.8	43.3	46.7	40.1	29.9	28.8	33.1	86.6	84.4	82.3
95% CI	90.4 to 92.6	90.7 to 93.3	84.0 to 86.6	52.9 to 66.5	53.3 to 66.6	50.8 to 60.8	40.8 to 45.9	44.3 to 49.1	38.3 to 42.0	23.5 to 36.3	22.8 to 34.8	28.0 to 38.2	83.9 to 89.3	81.7 to 87.1	79.6 to 85.0
P value for difference in means*	0.0000			0.0063			0.0000			0.0074			0.0000		
Proportion of households with tertiary level education (%)	71.8	69.7	78.5	38.7	36.5	37.6	51.2	44.6	39.7	30.9	22.5	17.2	35.7	28.5	32.6
95% CI	66.4 to 76.7	64.5 to 74.5	74.2 to 82.3	30.6 to 47.4	27.7 to 46.2	30.0 to 45.8	45.1 to 57.3	38.0 to 51.4	33.7 to 46.0	12.3 to 58.6	9.3 to 45.1	6.8 to 37.1	27.7 to 44.6	22.1 to 36.0	24.9 to 41.4
P value for difference in proportions†	0.0000			0.4771			0.0000			0.0139			0.0010		

*One-way analysis of variance test for difference in means.

†Wald test for difference in proportions.

HTN, hypertension; NCD, non-communicable disease; PURE, Prospective Urban and Rural Epidemiology.

**Figure 1:**
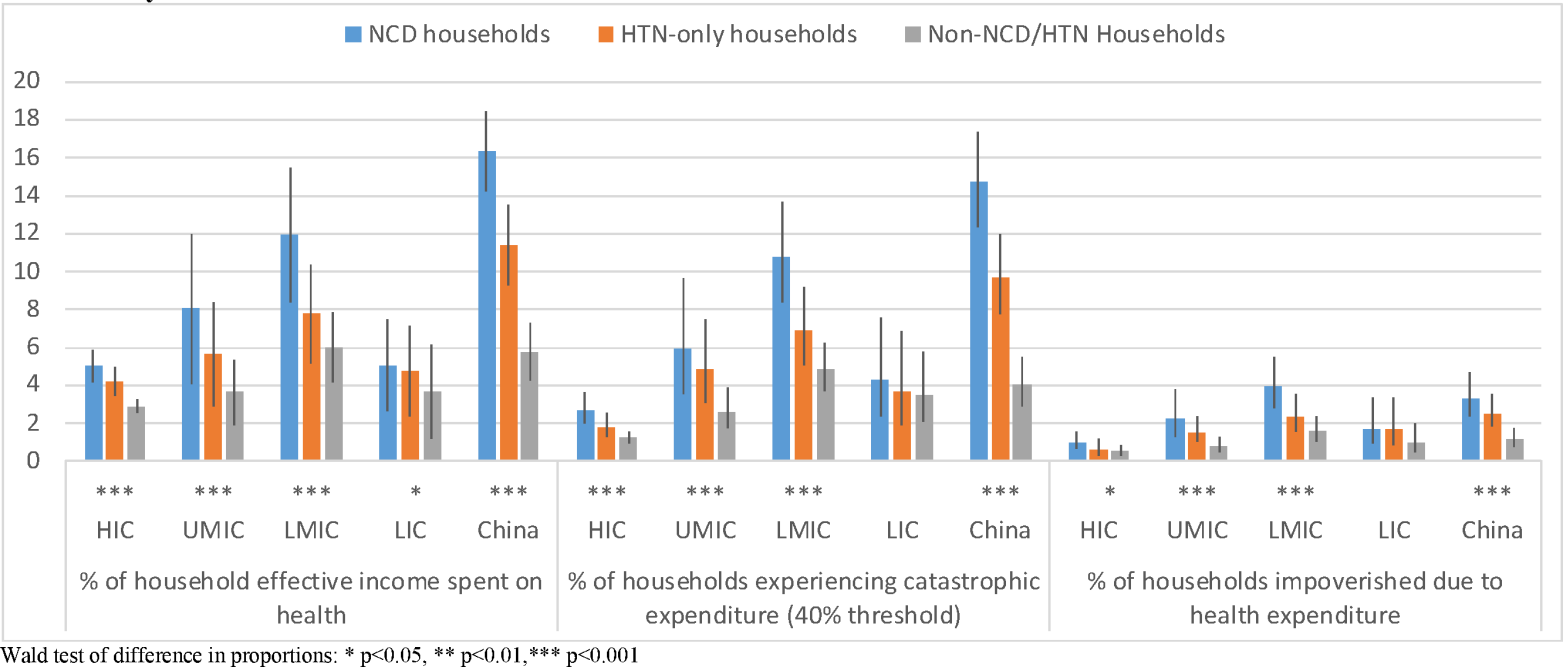
Economic burden of healthcare costs, comparing NCD, HTN only and non-NCD/HTN households by country income group, PURE Study. HIC, high-income country; LIC, lower income country; LMIC, lower middle income country; NCD, non-communicable disease; PURE, Prospective Urban and Rural Epidemiology; UMIC, upper middle income country.

Figure 1 shows the unadjusted comparison of mean household healthcare expenditure as a percentage of effective income, proportion of households experiencing catastrophic spending and proportion impoverished, across the three types of household. Households with at least one member with a NCD spend a higher percentage of their effective income on healthcare than HTN-only and non-NCD/HTN households, and differences are greatest in LMICs (NCD households: 12.0%; HTN only: 7.8%; non-NCD/HTN: 6.0%) and China (NCD households: 16.3%; HTN only: 11.4%; non-NCD/HTN: 5.8%). Catastrophic spending appears higher among NCD households in all country income groups apart from LICs and of impoverishment apart from HICs and LICs. Country specific results (online supplementary appendix 6) show highest percentages of catastrophic spending in Bangladesh (22.2%), Poland (21.9%) and Iran (14.7%) and the lowest in Malaysia (0.9%), Colombia (0.9%) and Turkey (1.8%). We also include a breakdown of average household monthly expenditure in NCD households by type of expenditure in online supplementary appendix table 7. In each country income group and China, the biggest driver of health expenditure was medicines, except for in LICs where it was diagnostics.

Table 2 shows the mean difference in percentage of effective income spent on healthcare, and the difference in risk of experiencing catastrophic spending and impoverishment, comparing NCD households or HTN households to non-NCD households, with 95% CIs, after adjusting for potentially confounding household characteristics. The presence of an NCD or HTN in a household increases the percentage of effective income spent on healthcare in LMICs, with NCD households spending on average 3.55% and HTN-only households 0.94% more than non-NCD households (p for trend <0.001) and in China (NCD households: 7.89%; HTN households: 4.24%; p<0.001). A similar pattern was observed in HICs, UMICs and LICs but with less dramatic increases. The risk of catastrophic spending from non-NCD/HTN to HTN only to NCD households increases significantly in all country income groups except for LICs, with the highest risk among NCDs households in China (risk difference: 7.52%). There is also a statistically significant increasing trend in risk of impoverishment in UMICs (risk difference for NCD households: 0.34%) and China (risk difference: 1.15%). As a sensitivity analysis, we reproduced table 2 using different country groupings by region (South Asia: India, Pakistan and Bangladesh; Canada/Sweden; South America: Brazil, Chile and Colombia; China; South East Asia: Malaysia and the Philippines; Middle East: Saudi Arabia, Iran, UAE and Turkey; and sub-Saharan Africa: South Africa, Tanzania and Zimbabwe). Results are shown in online supplementary appendix table 8. Our findings hold even with the different groupings, with NCD households at greater odds of catastrophic spending in all regions, with the highest odds experienced by NCD households in China, sub-Saharan Africa and South Asia.

**Table 2:** Difference in the percentage of effective income spent on healthcare, risk of experiencing catastrophic spending and risk of experiencing impoverishment, adjusted for household characteristics, NCD and HTN-only households compared with non-NCD/HTN households: PURE Study

Country income group	Households with no NCD/HTN		HTN-only versus non-NCD/HTN households		NCD versus non-NCD/HTN households		Test for trend*
**Percentage of effective income spent on health**							
	**N**	**%**	**Mean difference**	**95% CI**	**Mean difference**	**95% CI**	**P value**
High	7646	3.23	0.79	0.09 to 1.49	1.31	0.71 to 1.91	<0.001
Upper middle	13 741	4.75	0.34	−1.08 to 1.76	2.77	1.49 to 4.05	<0.001
Lower middle	9795	6.62	0.94	−1.66 to 3.53	3.55	1.31 to 5.80	0.001
Low	2155	3.07	0.98	−2.30 to 4.26	1.66	−1.27 to 4.59	0.269
China	18 489	7.07	4.24	3.16 to 5.32	7.89	6.85 to 8.94	<0.001
**Risk of catastrophic expenditure**							
	**N**	**%**	**Risk difference**	**95% CI**	**Risk difference**	**95% CI**	**P value**
High	7646	1.22	0.21	−0.50 to 0.92	0.77	0.09 to 1.45	0.018
Upper middle	13 741	1.36	0.29	−0.12 to 0.69	0.82	0.37 to 1.27	<0.001
Lower middle	9795	3.82	0.21	−0.76 to 1.19	1.71	0.75 to 2.67	<0.001
Low	2155	2.60	−0.27	−2.17 to 1.64	−0.22	−1.91 to 1.48	0.826
China	18 489	3.56	4.04	2.88 to 5.19	7.52	5.88 to 9.16	<0.001
**Risk of impoverishment**							
	**N**	**%**	**Risk difference**	**95% CI**	**Risk difference**	**95% CI**	**P value**
High	7646	0.42	0.01	−0.34 to 0.37	0.29	−0.08 to 0.66	0.0896
Upper middle	13 741	0.30	0.09	−0.06 to 0.25	0.34	0.13 to 0.55	<0.001
Lower middle	9795	1.10	−0.02	−0.43 to 0.39	0.43	0.01 to 0.84	0.062
Low	2155	0.65	0.81	(−0.38 to 2.01)	0.62	−0.30 to 1.54	0.446
China	18 489	0.82	0.66	0.28 to 1.04	1.15	0.66 to 1.63	<0.001

*Regression-based test substituting factor household variable (ie, NCD vs HTN-only vs non-NCD/HTN) with ordinal/linear variable, followed by likelihood ratio test to assess model fit.

HTN, hypertension; NCD, non-communicable disease; PURE, Prospective Urban and Rural Epidemiology.

Table 3 presents the proportion of individuals with NCDs and HTN reporting cost as a reason for not taking prescribed medicine, and reporting difficulty paying for various categories of healthcare costs, by gender. Each indicator was reported more commonly by women than men, in each country income group apart from in China, but numbers were very high for both genders. Most alarmingly, 38.7% of female NCD respondents in LICs reported not taking medicines in the last year because of cost, compared with 12.6% of males. Also, more than a third of female NCD and HTN respondents in LICs reported difficulties paying for every category of healthcare cost. These numbers were also very high in LMICs, where around a quarter of female NCD and HTN respondents reporting difficulty paying for medications.

**Table 3:** Self-reported forgone medication and difficulty paying for care due to costs, among women and men with NCDs and HTN: PURE Study (sample N’s represent those individuals from our included sample of households who responded to each question)

			High		Upper middle		Lower middle		Low		China	
			Female	Male	Female	Male	Female	Male	Female	Male	Female	Male
**Individuals with NCD**	**Did not take medications because of cost**	N	1787	1795	4638	2763	2311	1553	382	151	3076	2446
		%	4.0	2.5	6.1	3.7	17.0	14.0	38.7	12.6	1.1	1.2
		95% CI	2.5 to 6.4	(1.7 to 3.7)	4.3 to 8.6	2.6 to 5.3	13.5 to 21.3	11.2 to 17.3	24.9 to 54.7	4.9 to 28.9	(0.7 to 1.8	0.7 to 2.1
	**Difficulty paying doctor’s fees**	N	1453	1532	4202	2546	2222	1492	342	122	3015	2422
		%	2.7	1.6	8.2	5.7	23.8	19.2	34.2	14.8	3.8	3.8
		95% CI	1.9 to 3.7	1.1 to 2.5	5.2 to 12.7	3.5 to 9.1	18.8 to 29.6	15.4 to 23.6	23.6 to 46.6	5.1 to 36.0	2.2 to 6.6	2.2 to 6.4
	**Difficulty paying for medications**	N	1679	1712	4479	2694	2264	1531	348	123	3041	2435
		%	6.8	3.7	9.8	6.4	26.1	20.6	42.8	17.9	4.4	4.0
		95% CI	5.1 to 9.0	2.7 to 5.2	6.7 to 14.1	4.1 to 9.8	21.0 to 31.9	16.6 to 25.3	30.2 to 56.5	7.0 to 38.8	2.7 to 7.2	2.3 to 6.7
	**Difficulty paying diagnostics fees**	N	1454	1521	4093	2478	2168	1432	330	108	2975	2379
		%	1.5	1.1	7.2	5.2	21.7	17.8	34.2	18.5	5.1	4.7
		95% CI	1.0 to 2.3	0.6 to 1.9	4.2 to 12.2	3.1 to 8.5	17.2 to 26.9	14.2 to 22.1	24.8 to 45.1	7.0 to 40.9	3.2 to 8.0	2.9 to 7.3
	**Difficulty paying hospital bills**	N	960	1077	3329	2063	1406	912	328	113	2936	2360
		%	1.1	0.7	5.6	3.9	15.1	14.9	36.9	16.8	4.1	3.4
		95% CI	0.6 to 2.2	0.3 to 1.6	3.0 to 10.1	2.0 to 7.3	11.1 to 20.2	10.9 to 20.0	27.0 to 48.0	5.7 to 40.3	2.3 to 7.1	1.8 to 6.4
**Individuals with HTN only**	**Did not take medications because of cost**	N	1226	1173	3734	1989	1685	910	348	105	3164	2553
		%	1.8	1.4	4.7	2.5	13.3	8.2	34.8	26.7	1.5	1.1
		95% CI	1.2 to 2.6	0.8 to 2.4	3.4 to 6.5	1.8 to 3.5	10.0 to 17.4	6.3 to 10.7	24.8 to 46.3	13.3 to 46.4	0.9 to 2.5	0.6 to 1.8
	**Difficulty paying doctor's fees**	N	1011	997	3292	1749	1575	848	308	91	3080	2500
		%	1.3	0.7	8.7	6.0	18.1	13.0	39.3	22.0	3.4	2.1
		95% CI	0.7 to 2.2	0.3 to 1.4	5.8 to 13.1	3.9 to 9.1	14.0 to 23.0	9.7 to 17.1	27.1 to 53.0	8.7 to 45.3	2.0 to 5.6	1.3 to 3.5
	**Difficulty paying for medications**	N	1176	1135	3579	1899	1628	885	315	95	3124	2527
		%	2.3	1.9	8.4	5.7	20.3	15.3	39.7	22.1	3.5	2.5
		95% CI	1.4 to 3.8	1.2 to 3.0	5.7 to 12.0	3.8 to 8.5	16.0 to 25.5	11.7 to 19.7	31.0 to 49.0	10.0 to 41.9	2.0 to 6.0	1.5 to 4.3
	**Difficulty paying diagnostics fees**	N	992	1004	3236	1697	1499	789	299	92	3023	2456
		%	0.6	0.6	7.1	5.5	16.9	10.4	37.5	25.0	4.0	2.9
		95% CI	0.3 to 1.3	0.3 to 1.3	4.6 to 10.7	3.4 to 8.9	13.2 to 21.3	7.6 to 14.0	26.4 to 50.0	11.9 to 45.2	2.3 to 6.7	1.6 to 5.3
	**Difficulty paying hospital bills**	N	591	602	2642	1337	954	470	296	88	3006	2439
		%	1.2	0.7	7.1	5.2	9.7	8.7	36.8	22.7	2.7	1.4
		95% CI	0.6 to 2.4	0.2 to 1.8	3.9 to 12.5	3.0 to 8.7	6.9 to 13.7	5.8 to 12.9	25.6 to 49.7	10.2 to 43.3	1.4 to 5.2	0.8 to 2.4

HTN, hypertension; NCDs, non-communicable diseases.

Table 4 shows the proportion of individuals who have NCDs and HTN (and who responded to this question) reporting four types of coping strategies. Reimbursement from insurance was not included as, in that case, the individual or their family were not making the payment. While using income or savings was the most common in all country income groups, borrowing money from family or friends also appears important in LICs (NCD respondents: 12.8%; HTN only: 7.6%) and in LMICs (NCD respondents: 10.1%; HTN only: 7.0%). In LICs, selling items was reported by 5.9% of NCD respondents and 11.3% of HTN-only respondents.

**Table 4:** Sources of financing reported by individuals with NCDS and HTN to pay for healthcare expenditures: PURE Study

Participant group	Country income group	Used income/savings			Sold items (eg, land, property and jewellery)			Borrowed from relatives or friends			Borrowed from financial institutions		
		N	%	95% CI	N	%	95% CI	N	%	95% CI	N	%	95% CI
**Individuals with NCD**	High	3758	77.9	74.0 to 81.4	3679	0.6	0.4 to 1.0	3680	0.8	0.4 to 1.4	3678	1.8	1.1 to 2.8
	Upper middle	7696	58.1	46.9 to 68.5	7488	0.5	0.3 to 0.8	7488	1.9	1.1 to 3.2	7488	0.6	0.4 to 0.9
	Lower middle	4007	77.3	70.9 to 82.6	3909	1.4	0.9 to 2.2	3909	10.1	7.4 to 13.6	3908	2.4	1.5 to 3.6
	Low	557	78.5	61.0 to 89.5	508	5.9	2.7 to 12.3	508	12.8	4.9 to 29.4	508	0.2	0.0 to 1.7
	China	5861	81.4	69.3 to 89.4	5520	2.2	1.0 to 4.6	5520	2.8	1.4 to 5.4	5520	0.6	0.3 to 1.2
**Individuals with HTN only**	High	2422	81.8	78.2 to 84.9	2387	0.8	0.5 to 1.3	2387	0.5	0.3 to 0.9	2387	1.7	1.1 to 2.6
	Upper middle	5912	54.4	42.1 to 66.2	5758	0.6	0.3 to 1.0	5758	1.6	0.8 to 3.1	5758	0.5	0.3 to 0.8
	Lower middle	2712	71.1	62.6 to 78.3	2640	1.1	0.7 to 1.7	2640	7.0	5.2 to 9.3	2640	1.6	1.0 to 2.5
	Low	469	71.4	59.5 to 81.0	432	11.3	6.0 to 20.5	432	7.6	3.6 to 15.3	432	0.5	0.1 to 2.1
	China	5960	85.4	78.3 to 90.5	5501	2.7	1.4 to 5.1	5501	3.1	1.5 to 6.5	5501	0.9	0.4 to 1.9

HTN, hypertension; NCDs, non-communicable diseases.

## Discussion

The primary goal of the 2014 UN commitments on NCDs is to protect people from dying prematurely from heart and lung diseases, cancers and diabetes. To achieve this aim, the WHO Global Action Plan for the Prevention and Control of NCDs stresses the importance of ensuring affordable access to early diagnosis and treatment for those with NCDs.^27^ Our paper represents the first multicountry estimates since 2014 of the burden of healthcare costs faced by households with NCDs, using current global standards of economic burden measurement and a consistent approach across a sample of countries at different levels of development. Our analysis shows that we are far from realising this goal; households with NCDs and HTN experience a higher burden of healthcare than those without NCDs, and in LICs, the burden may be disguised by foregone care.

After adjusting for factors that might drive household health expenditure, we find that households with NCDs experience statistically significantly higher risk of catastrophic spending than non-NCD/HTN households in all country income groups except for LICs, with the highest risk in LMICs and China. They also have statistically significantly higher risk of impoverishment in UMICs, LMICs and China. Greater risk is also observed in households with HTN only, but these are more modest. This may be because HTN is a silent condition often untreated and not incurring expenditures. Indeed only 6.5% of people with HTN in African PURE countries and only 18.4% in North American and European PURE countries have their HTN controlled.^28^ Treatment of HTN is also inherently less expensive than treatment for most NCDs, which usually requires multiple medications and more frequent visits to the health facility. This likely explains the lower financial risk in the HTN group. This also emphasises the benefits of intervening early with HTN to prevent complications and to reduce the economic burden.

While the higher economic burden in LMICs compared with LICs may appear surprising, this is consistent with other evidence that people initially pay more OOP as countries become richer, with OOP only falling again as they progress to UMICs and HICs.^29^ In the poorest countries, households are more likely to forgo care because they simply cannot afford it. This, paradoxically, reduces catastrophic spending and impoverishment on care.^30^

In LICs, patients with NCDs may finance healthcare expenditures by borrowing from family or friends, as we show here (12.8% of people). This is intuitive as many households in these countries will be unable to pay large sums from current income or savings,^31 32^ thereby causing them to seek alternative strategies.^32 33^ Although borrowing may allow households to pay for care for short periods, it can conceal potential longer term economic consequences when loans need to be repaid,^33^ or impact on treatment adherence when healthcare costs are chronic and recurring, as in the case of NCDs. As such, cross-sectional studies are limited in their capacity to estimate the true economic impact of healthcare costs on households who employ such coping strategies in the short term.^34^ It is important to consider how households finance OOP health expenditures when designing methods to monitor financial risk protection.^32^

The impact that OOP can have on treatment adherence is evident in our findings, and this appears to affect women more than men in LICs. More than 30% of women in LIC reported that they did not take medications in the last year due to cost and that they had difficulty paying for doctor’s fees, medications, diagnostic fees and hospital bills. This may in part explain why fewer women are using effective treatment for conditions like CVD.^12^ Research from several African countries has shown that women are more likely than men in the highest income group to use healthcare but that the reverse is true in the lowest income groups.^35^ In households where the male is the sole breadwinner, his falling ill is likely to have a larger impact on income than when a woman falls ill.^36^ Consequently, healthcare of male members of the household may be prioritised over females. However, other factors play a part, including lower literacy,^37^ less autonomy,^38^ less disposable income^39^ and less control over household financial resources.^40^ This gender gap emphasises the urgency of UHC as a pathway to equity in health.

### Limitations

Earlier work has suggested that health-focused surveys, such as the WHO World Health Survey^41^ from which the PURE Study expenditure module was adapted, which include only brief modules on general (ie, non-health care) expenditure, can overestimate the burden of healthcare expenditure on households,^42^ due to some general expenditure items being missed or the participants’ attention being drawn more to their health expenditure. The PURE Study may be vulnerable in this way, and our figures for catastrophic spending and impoverishment among NCD households are higher than previous findings from the general population.^43 44^ However, if we are indeed underestimating general expenditure, this would affect NCD, HTN and non-NCD households equally, so this should not affect our estimates of the differences in catastrophic spending or impoverishment.

There is substantial debate about the most appropriate measures of catastrophic spending and impoverishment,^22^ and our choice for the main analyses may be criticised by proponents of alternative approaches. However, online supplementary appendix 5 presents all the alternatives used in the WB/WHO UHC Tracking Report, and while the observed magnitude of each indicator differs, as expected, the pattern is the same, revealing a consistently higher burden among NCD households versus non-NCD households, particularly in LMICs. Regardless of the measure used, our estimates of economic burden are limited in that they include only one aspect of economic burden, that is, healthcare expenditures, and do not capture the indirect burden caused by loss of productivity and wages due to ill health.

Our analysis relied on self-reported health data, which can be subject to reporting bias. However, in the PURE Study, self-reported information on death, myocardial infarction, stroke, heart failure, cancer, hospitalisations, new diabetes, chronic obstructive pulmonary disease and several other acute conditions was adjudicated centrally in each country by trained physicians using standardised definitions. For example, self-reported CVD events were verified against medical or hospital records in 455 reported events, with a confirmation rate of 89%. Further follow-up has shown that mortality among those with self-reported coronary heart disease or stroke is 4–6 times that of the population without, serving as a measure of the validity of the self-reports. With respect to HTN, the accuracy of self-reported diagnosis has been shown by a number of studies, and the use of self-reported HTN treatment or diagnosis to define HTN prevalence is supported by the World Hypertension League.^45^ Our analysis includes many of the most common NCDs; it does not include the full range of NCDs that cause financial hardship for patients’ households. The healthcare costs associated with other NCDs, for example, mental disorders, is also likely to contribute to the financial burden faced by households.^46^

Finally, the PURE Study’s design as a cohort study necessitated inclusion of subjects in communities who could feasibly and affordably be followed up over many years. As such, the sampling framework in each country was not designed to be nationally representative. However, the PURE Study household sample has showed good concordance with the national age, sex, place of residence (urban/rural), education and mortality profiles of the study countries, suggesting that there was no systemic bias in data collection.^16^ As mentioned above, further information on the representativeness of the PURE Study cohort is included as online supplementary appendix 1.

## Conclusion

Our findings show that we are far from achieving financial risk protection for people with NCDs, particularly in LMICs and LICs. While the burden of NCD care may appear greatest in LMICs, the burden in LICs may be disguised by foregone care due to costs. Females with NCDs are most markedly affected by OOP for NCD care, contributing to gender inequality in treatment. At a time when the international community is reviewing progress towards mutually agreed NCD goals, the data summarised in this paper highlight the need for urgent solutions, taking a gendered perspective, to make NCD care more accessible to all.

## Data Availability

Data may be obtained from a third party and are not publicly available. Participant-level data cannot be publicly deposited because consent to share individuals’ data publicly has not been obtained, and data collection is ongoing. Researchers wishing to access these data should contact the PURE Program Manager, Sumathy Rangarajan at sumathy.rangarajan@phri.ca.
