# Supplementary material for: The household economic burden of non-communicable diseases in 18 countries
Source: BMJ Glob Health. 2020 Feb 11;5(2):e002040. doi: 10.1136/bmjgh-2019-002040 (PMC7042605; doi:10.1136/bmjgh-2019-002040)
Supplement: Supplementary data [file bmjgh-2019-002040supp001.pdf]

Appendices

Appendix 1: Representativeness of the PURE Cohort (1)

a) Are the countries included in the PURE cohort atypical?

We compared the countries participating in PURE with those participating in MONICA,(2) the largest previous study of this nature conducted. We plotted national income (GDP/capita) against research output (number of publications recorded in SCOPUS between 1996 and 2010 per 100,000 population). These data are taken from a previous study undertaken by one of the authors (M.McKee) on global health research capacity to inform policy discussions within WHO. (3) These graphs (Figures S1 and S2) demonstrate that PURE has captured the full diversity of countries on these two dimensions (Figure S1), in marked contrast to MONICA, which was concentrated in high income countries with substantial research capacity (Figure S2).

b) Are PURE populations representative of the countries in which they are situated?

The PURE household population compared to national statistics had more women (sex ratio 95.1 men per 100 women vs 100.3) and was older (33.1 years vs 27.3), although age had a positive linear relationship between the two data sources (Pearson's r = 0.92). PURE was 59.3% urban compared to an average of 63.1% in participating countries. The distribution of education was less than 7% different for each category, although PURE households typically had higher levels of education. For example, 37.8% of PURE household members had completed secondary education compared to 31.3% in the national data. However, age-adjusted annual mortality rates showed positive correlation for men (r = 0.91) and women (r = 0.92) but were lower in PURE compared to national statistics (7.9 per 1000 vs 8.7 for men; 6.7 vs 8.1 for women) (Figure S3).

These findings indicate that modest differences exist between the PURE household population and national data for the indicators studied. These modest differences, however, are unlikely to have much influence on exposure-disease (or health systems assessments vs outcome) associations derived in PURE. (4)

Figure S1: Countries Participating in PURE by National Income and Medical Research Capacity

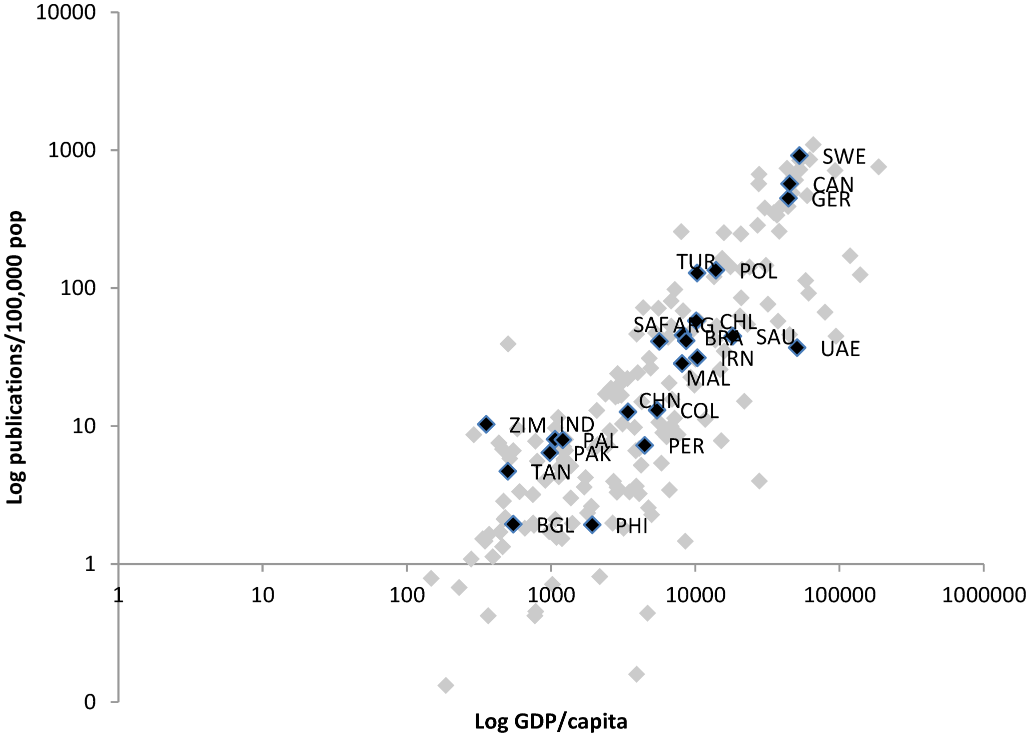

Figure S2: Countries Participating in MONICA by National Income and Medical Research Capacity

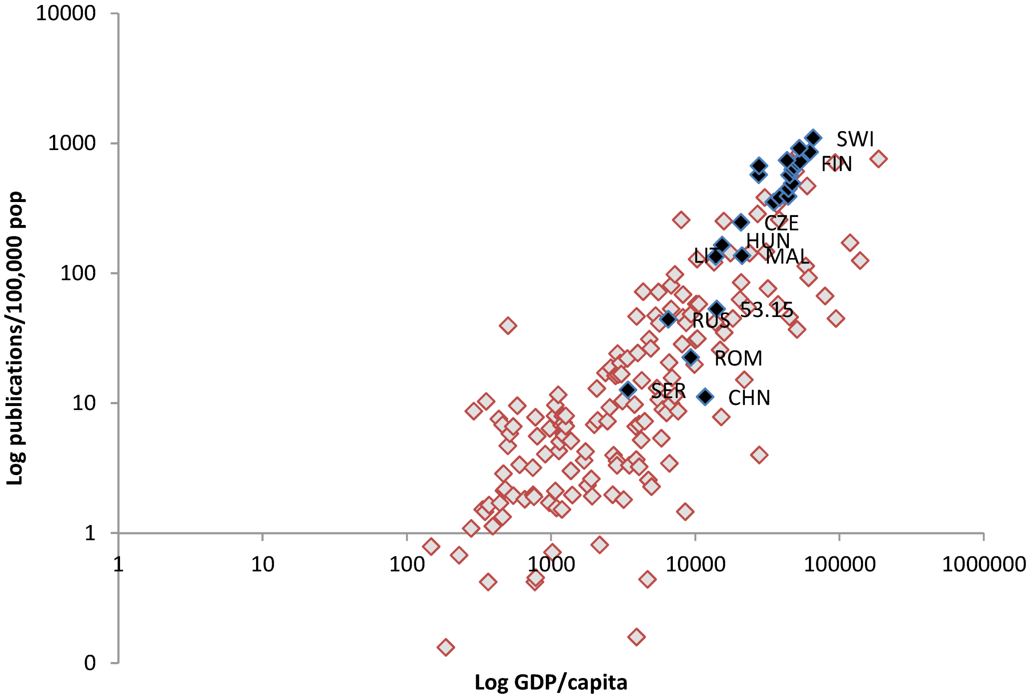

For clarity, only a few of the richest and most productive countries in the MONICA study are labeled in this graph. However, all of the middle-income or lower-income countries, and those with lower levels of research productivity, are labeled.

Figure S3: Mortality in PURE Households Reported in the Previous Two Years Versus National Mortality Data[5]

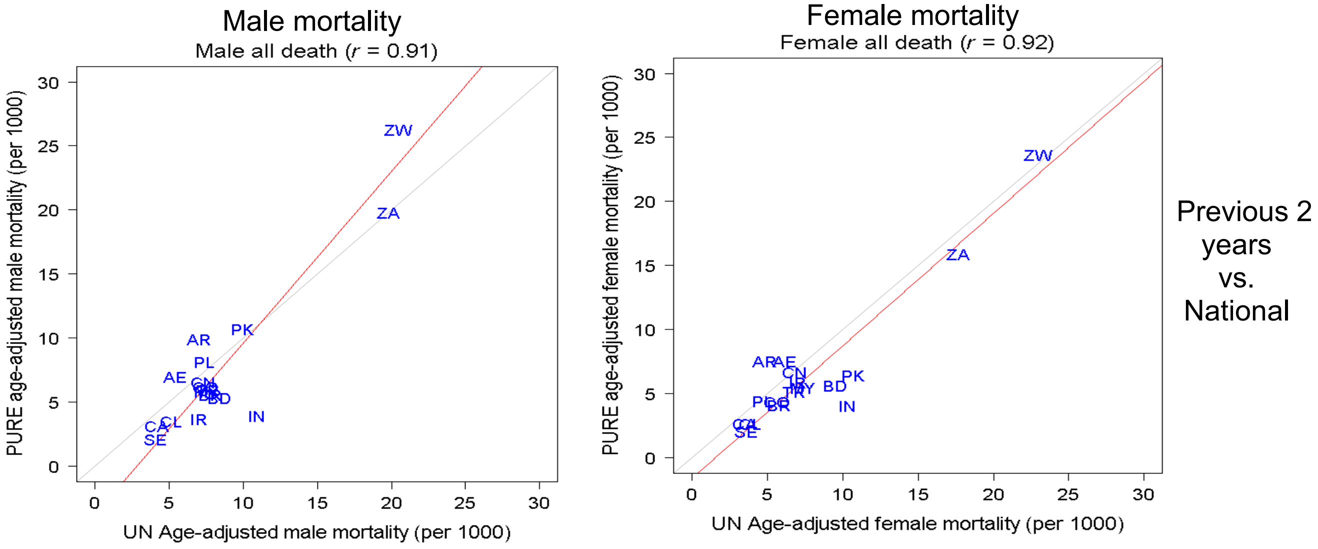

Appendix Table 2: Table of follow-up data collection dates

Country	Start date	End date
Bangladesh	Sep-2015	Nov-2015
Brazil	Jul-2014	Jun-2018
Canada	Jan-2014	Jun-2018
Chile	Apr-2014	May-2018
China	May-2014	Feb-2018
Colombia	July-2014	May-2018
India	May-2014	Mar-2018
Iran	May-2014	Feb-2018
Malaysia	May-2014	May-2018
Occupied Palestinian Territory	Jan-2015	Apr-2017
Pakistan	Oct-2014	Sep-2016
Philippines	Feb-2016	Feb-2018
Poland	Sep-2014	Jun-2018
South Africa	Jan-2014	Sep-2017
Sweden	May-2014	May-2018
Tanzania	Nov-2015	Jun-2018
Turkey	May-2014	Dec-2017
Zimbabwe	Dec-2014	Jan-2017

Appendix Table 3: Table of respondents who self-reported a CVD, cancer, COPD, asthma, diabetes or hypertension at baseline but not at follow-up (%)

Country	Total number of individuals	Self-reported hypertension or diabetes at baseline but not at follow up		Self-reported CVD, cancer, COPD or asthma baseline but not at follow up	
	N	n	%	n	%
Bangladesh	56	2	3.6%	0	0.0%
Brazil	4075	42	1.0%	0	0.0%
Canada	9290	99	1.1%	7	0.1%
Chile	2166	44	2.0%	0	0.0%
China	28406	673	2.4%	0	0.0%
Colombia	3370	86	2.6%	0	0.0%
India	3767	67	1.8%	0	0.0%
Iran	5631	2	0.0%	0	0.0%
Malaysia	7974	224	2.8%	0	0.0%
Occupied Palestinian Territory	1378	25	1.8%	0	0.0%
Pakistan	1272	57	4.5%	0	0.0%
Philippines	152	3	2.0%	0	0.0%
Poland	3465	30	0.9%	0	0.0%
South Africa	2246	54	2.4%	1	0.0%
Sweden	4015	36	0.9%	39	1.0%
Tanzania	820	19	2.3%	0	0.0%
Turkey	4657	52	1.1%	0	0.0%
Zimbabwe	719	19	2.6%	0	0.0%

Appendix Table 4: Table comparing characteristics of households that did and did not complete the expenditure questionnaire*

Country income group	Number of households			Median highest level of education achieved in household			Mean number of household members			Mean % of female household members		
	Responders	Non-responders	Lost to follow up	Responders	Non-responders	Lost to follow up	Responders	Non-responders	Lost to follow up	Responders	Non-responders	Lost to follow up
High	7,646	1,625	471	Tertiary	Tertiary	Tertiary	2.3	1.4	6.9	52.7%	46.8%	47.7%
Upper-middle	13,741	3,349	312	Secondary	Secondary	Tertiary	3.8	3.9	2.9	53.1%	48.2%	56.9%
Lower-middle	9,795	13,080	2,058	Secondary	Secondary	Tertiary	4.0	5.2	5.1	50.5%	51.4%	55.0%
Low	2,155	1,965	658	Secondary	Secondary	Primary	5.4	4.8	4.7	53.0%	40.6%	50.7%
China	18,489	5,160	71	Secondary	Secondary	Secondary	2.9	3.5	3.2	50.8%	49.5%	44.3%

Country income group	Mean % of household members over 60 years old			Mean % of household members under 5 years old			Mean age of household members		
	Responders	Non-responders	Lost to follow up	Responders	Non-responders	Lost to follow up	Responders	Non-responders	Lost to follow up
High	16.3%	17.6%	1.3%	0.7%	0.8%	1.3%	45.9	46.8	27.3
Upper-middle	9.0%	8.8%	16.2%	0.8%	0.7%	0.6%	39.5	39.5	46.7
Lower-middle	5.3%	4.5%	6.5%	0.9%	1.0%	1.2%	35.0	32.7	35.7
Low	3.7%	4.8%	5.6%	1.3%	1.2%	1.2%	30.5	30.8	32.2
China	8.9%	10.1%	12.3%	0.4%	0.5%	0.1%	44.1	43.4	42.2

*Three countries that are included in the PURE study were not included in this analysis: Argentina, Saudi Arabia and the United Arab Emirates. Expenditure data were not collected in Argentina at follow up due to practical reasons and concerns regarding participant burden. In Saudi Arabia and UAE, while the expenditure section was used, the vast majority of followed up households did not provide the data needed to derive our financial risk indicators (e.g. missing data on food, rent), thus limiting our ability to conduct any meaningful analyses in these countries.

**High-income: Canada, Sweden; Upper-middle income: Brazil, Chile, Malaysia, Poland, South Africa, Turkey; Lower-middle income: the Philippines, Colombia, India, Iran, the Occupied Palestinian Territory; Low-income: Bangladesh, Pakistan, Zimbabwe, Tanzania.

The table above suggests that there were no notable differences between households that did or did not respond to the expenditure questionnaire. There are some small differences between households that were lost to follow up and those that were not. For example, these households were younger in HIC, and better educated in UMIC and LMIC, but less educated in LIC. It is possible that these characteristics have an influence on whether household members are home when field workers visit or are more likely to move house between baseline and follow up.

Appendix Table 5: Households immiserised (i.e. already impoverished and pushed further into poverty) by OOP on health (%)

For those households where actual total expenditure is lower than the basic needs line, the denominator (total expenditure less basic needs line) will be negative. Rather than using these households' actual spending on basic needs,(5) we allow the denominator to remain negative to capture 'immiserising' OOP expenditure, a concept introduced by Wagstaff and Eozenou (6) to highlight cases where households already in poverty are pushed further into poverty by OOP on health. The proportion of households immiserised is presented in Appendix 4.

Country	Total number of households	Households immiserated	
	N	n	%
Bangladesh	37	4	10.8
Brazil	2937	451	15.4
Canada	5268	679	12.9
Chile	1304	339	26.0
China	18489	3284	17.8
Colombia	2467	589	23.9
India	2295	449	19.6
Iran	3582	573	16.0
Malaysia	4521	1120	24.8
Occupied Palestinian Territory	1362	146	10.7
Pakistan	831	180	21.7
Philippines	89	15	16.9
Poland	1342	147	11.0
South Africa	1554	289	18.6
Sweden	2378	489	20.6
Tanzania	683	152	22.3
Turkey	2083	322	15.5
Zimbabwe	604	139	23.0

Appendix 6: Table of country-specific results of main outcomes

	Mean total per capita household expenditure on health care (Int $)								
	NCD households			HTN-only households			Non-NCD/HTN Households		
**Country**	**N**	**Mean**	**(95% CI)**	**N**	**Mean**	**(95% CI)**	**N**	**Mean**	**(95% CI)**
Bangladesh	9	14.4	(-4.6-33.4)	6	8.7	(-11.9-29.4)	22	55.4	(12.6-98.1)
Brazil	1196	12.4	(6.7-18.2)	801	6.1	(2.9-9.3)	940	5.6	(2.3-9.0)
Canada	2046	5.5	(4.4-6.5)	942	5.0	(3.9-6.1)	2280	3.3	(2.9-3.7)
Chile	561	5.2	(2.1-8.4)	312	5.3	(1.6-9.0)	431	4.4	(2.2-6.5)
China	5249	16.3	(14.2-18.4)	4446	11.4	(9.3-13.5)	8794	5.8	(4.3-7.3)
Colombia	565	1.9	(1.3-2.5)	598	1.9	(0.9-2.8)	1304	1.1	(0.7-1.5)
India	950	7.8	(4.6-11.0)	434	4.9	(0.9-8.8)	911	3.1	(0.9-5.2)
Iran	1444	18.9	(12.0-25.8)	723	13.2	(7.8-18.5)	1415	11.0	(6.6-15.4)
Malaysia	1808	0.9	(0.6-1.3)	981	0.6	(0.2-1.0)	1732	0.6	(0.3-0.8)
Occupied Palestinian Territory	441	11.4	(8.9-13.9)	172	9.3	(4.7-14.0)	749	9.0	(7.0-11.0)
Pakistan	253	3.3	(-2.7-9.2)	101	5.0	(-6.8-16.8)	477	3.7	(-5.1-12.5)
Philippines	29	7.7	(3.4-12.0)	21	37.9	(-23.8-99.7)	39	2.0	(-1.8-5.9)
Poland	606	33.2	(16.9-49.5)	408	21.7	(20.2-23.1)	328	18.4	(17.1-19.7)
South Africa	324	6.0	(1.0-11.1)	570	5.1	(1.3-9.0)	660	3.2	(1.9-4.5)
Sweden	714	3.7	(2.8-4.7)	565	3.0	(2.3-3.6)	1099	2.1	(1.7-2.5)
Tanzania	110	8.1	(4.4-11.7)	127	3.8	(-1.3-8.9)	446	2.9	(0.7-5.2)
Turkey	964	2.4	(1.8-3.0)	441	2.2	(1.2-3.3)	678	1.6	(1.2-2.0)
Zimbabwe	143	5.3	(-2.9-13.5)	175	5.1	(-1.0-11.2)	286	0.8	(-0.4-2.1)

	Percentage experiencing catastrophic expenditure (40% threshold)								
	NCD households			HTN-only households			Non-NCD/HTN Households		
**Country**	**N**	**%**	**(95% CI)**	**N**	**%**	**(95% CI)**	**N**	**%**	**(95% CI)**
Bangladesh	9	22.2	(3.4-69.8)	6	0.0	(0.0-0.0)	22	27.3	(14.7-44.9)
Brazil	1196	10.6	(5.1-20.9)	801	7.7	(3.8-15.0)	940	5.0	(2.2-10.8)
Canada	2046	2.9	(2.1-4.0)	942	2.3	(1.6-3.3)	2280	1.2	(0.9-1.6)
Chile	561	2.5	(1.1-5.8)	312	3.2	(0.9-11.2)	431	2.1	(1.2-3.8)
China	5249	14.7	(12.3-17.4)	4446	9.7	(7.8-12.0)	8794	4.0	(2.9-5.5)
Colombia	565	0.9	(0.4-2.1)	598	2.2	(1.1-4.2)	1304	0.8	(0.5-1.5)
India	950	8.6	(6.2-11.9)	434	4.8	(2.5-9.1)	911	3.4	(1.9-5.9)
Iran	1444	16.8	(12.6-22.1)	723	11.9	(8.5-16.5)	1415	8.9	(6.7-11.8)
Malaysia	1808	0.9	(0.5-1.7)	981	0.4	(0.1-1.1)	1732	0.7	(0.4-1.3)
Occupied Palestinian Territory	441	8.6	(6.1-12.1)	172	6.4	(3.4-11.8)	749	6.0	(4.1-8.8)
Pakistan	253	2.4	(0.5-9.8)	101	5.0	(0.6-29.5)	477	4.2	(0.9-17.3)
Philippines	29	3.4	(1.7-6.7)	21	14.3	(3.8-41.2)	39	0.0	(0.0-0.0)
Poland	606	21.9	(11.1-38.8)	408	15.4	(12.5-18.9)	328	10.4	(5.0-20.2)
South Africa	324	4.9	(1.9-12.0)	570	4.0	(1.7-9.3)	660	2.3	(1.1-4.4)
Sweden	714	2.1	(1.2-3.8)	565	0.9	(0.4-2.0)	1099	1.2	(0.6-2.3)
Tanzania	110	6.4	(4.9-8.2)	127	2.4	(0.2-20.1)	446	3.1	(1.3-7.5)
Turkey	964	1.8	(1.0-3.0)	441	1.6	(0.6-4.4)	678	1.2	(0.7-2.0)
Zimbabwe	143	4.9	(0.2-63.5)	175	4.0	(0.6-22.3)	286	1.0	(0.2-5.0)

	Percentage impoverished								
	NCD households			HTN-only households			Non-NCD/HTN Households		
**Country**	**N**	**%**	**(95% CI)**	**N**	**%**	**(95% CI)**	**N**	**%**	**(95% CI)**
Bangladesh	9	0.0	(0.0-0.0)	6	0.0	(0.0-0.0)	22	13.6	(5.6-29.5)
Brazil	1196	4.8	(1.9-11.3)	801	3.1	(1.6-6.2)	940	2.1	(0.8-5.6)
Canada	2046	1.1	(0.7-1.9)	942	0.8	(0.4-1.8)	2280	0.4	(0.2-0.7)
Chile	561	0.5	(0.3-1.0)	312	0.6	(0.0-9.6)	431	0.2	(0.0-3.5)
China	5249	3.3	(2.3-4.7)	4446	2.5	(1.8-3.5)	8794	1.2	(0.8-1.8)
Colombia	565	0.4	(0.1-1.4)	598	0.5	(0.2-1.6)	1304	0.3	(0.1-0.8)
India	950	2.7	(1.8-4.2)	434	2.1	(0.8-5.4)	911	1.3	(0.6-2.7)
Iran	1444	7.2	(5.0-10.2)	723	4.1	(2.6-6.5)	1415	3.5	(2.1-5.7)
Malaysia	1808	0.2	(0.1-0.5)	981	0.3	(0.1-1.2)	1732	0.1	(0.0-0.4)
Occupied Palestinian Territory	441	0.7	(0.2-2.0)	172	0.6	(0.1-4.3)	749	0.7	(0.2-1.8)
Pakistan	253	1.2	(0.1-9.5)	101	2.0	(0.3-10.6)	477	1.0	(0.1-8.5)
Philippines	29	0.0	(0.0-0.0)	21	14.3	(3.8-41.2)	39	0.0	(0.0-0.0)
Poland	606	7.4	(2.5-20.2)	408	3.4	(1.9-6.2)	328	2.4	(1.0-5.7)
South Africa	324	2.8	(0.8-9.5)	570	1.4	(0.5-3.9)	660	0.9	(0.4-1.9)
Sweden	714	0.7	(0.2-2.1)	565	0.2	(0.0-1.3)	1099	0.8	(0.4-1.9)
Tanzania	110	3.6	(1.8-7.3)	127	1.6	(0.2-14.0)	446	0.7	(0.1-4.9)
Turkey	964	0.4	(0.1-1.2)	441	0.7	(0.2-2.0)	678	0.0	(0.0-0.0)
Zimbabwe	143	1.4	(0.1-15.4)	175	1.7	(0.2-13.3)	286	0.3	(0.0-9.6)

Appendix Table 7: Sensitivity analysis of catastrophic spending using regional country groupings

**Sensitivity analysis**	Adjusted odds ratio of experiencing catastrophic expenditure								
		NCD		HTN only		Non-NCD/Risk factor		Trend
Country income group	N	aOR	(95% CI)	aOR	(95% CI)	aOR	(95% CI)	p-value
South Asia	3160	1.76	(1.18-2.63)	1.12	(0.65-1.92)	1.00	(ref)	0.003
China	18487	3.37	(2.90-3.92)	2.22	(1.89-2.62)	1.00	(ref)	0.000
South East Asia	4296	1.54	(0.66-3.59)	1.23	(0.44-3.47)	1.00	(ref)	0.308
Africa	2819	2.94	(1.52-5.68)	2.12	(1.09-4.16)	1.00	(ref)	0.002
N America/Europe	11071	1.57	(1.17-2.11)	1.20	(0.86-1.68)	1.00	(ref)	0.001
Middle/Near East	4944	1.28	(1.01-1.63)	0.90	(0.66-1.21)	1.00	(ref)	0.017
South America	6708	1.55	(1.10-2.19)	1.23	(0.85-1.79)	1.00	(ref)	0.009

1. Yusuf S, Rangarajan S, Teo K, Islam S, Li W, Liu L, et al. Cardiovascular risk and events in 17 low-, middle-, and high-income countries. N Engl J Med. 2014;371(9):818-27.
2. Tunstall-Pedoe H, Vanuzzo D, Hobbs M, Mahonen M, Cepaitis Z, Kuulasmaa K, et al. Estimation of contribution of changes in coronary care to improving survival, event rates, and coronary heart disease mortality across the WHO MONICA Project populations. Lancet. 2000;355(9205):688-700.
3. McKee M, Stuckler D, Basu S. Where there is no health research: what can be done to fill the global gaps in health research? PLoS Med. 2012;9(4):e1001209.
4. Corsi DJ, Subramanian SV, Chow CK, McKee M, Chifamba J, Dagenais G, et al. Prospective Urban Rural Epidemiology (PURE) study: Baseline characteristics of the household sample and comparative analyses with national data in 17 countries. Am Heart J. 2013;166(4):636-46.e4.
5. World health Organization. WHO World Health Survey 2002 [cited Feb 8 2018]. Available from: http://www.who.int/healthinfo/survey/en/.
6. Wagstaff A, Eozenou PH. CATA meets IMPOV: A unified approach to measuring financial protection in health. Policy Research Working Paper 6861. The World Bank, Development Research Group, Human Development and Public Services Team; 2014.
